# EAST MEETS WEST: PERSPECTIVES ON CAREGIVING, FRAILTY, AND BASIC RESEARCH ON AGING IN CHINA AND THE UNITED STATES

**DOI:** 10.1093/geroni/igae098.1472

**Published:** 2024-12-31

**Authors:** James Nelson, Jianye Wang

**Affiliations:** Chair; Co-Chair

## Abstract

This interdisciplinary symposium brings together scholars from China and the United States to share recent discoveries from the health and biological sciences, as well as public policy initiatives designed to increase accessibility to established means of promoting health and well being of older individuals. Included will be discussions of novel therapeutics that may alleviate the pathophysiological burdens of senescence. An overarching premise of this cross-cultural symposium is that the unique cultural and experiential perspectives of scientists and practitioners from these two countries can enrich the approaches to research, education, and practice of geroscience and gerontology in both countries. Another goal is to build new bridges of communication between the two countries to promote collaboration, thereby synergizing research and practice everywhere. To this end, presentations on frailty and novel approaches to increase access to known behavioral interventions bring together different methodological perspectives, as will presentations on basic biology of vascular aging and pre-clinical pharmacologic interventions informing clinical trials aimed at restoring physiological functions in older individuals.

